# Predicting negative attitudes towards suicide in social media texts: prediction model development and validation study

**DOI:** 10.3389/fpubh.2024.1401322

**Published:** 2024-07-08

**Authors:** Ang Li

**Affiliations:** Department of Psychology, Beijing Forestry University, Beijing, China

**Keywords:** suicide, public attitudes, LIWC, machine learning, external validation

## Abstract

**Background:**

Implementing machine learning prediction of negative attitudes towards suicide may improve health outcomes. However, in previous studies, varied forms of negative attitudes were not adequately considered, and developed models lacked rigorous external validation. By analyzing a large-scale social media dataset (Sina Weibo), this paper aims to fully cover varied forms of negative attitudes and develop a classification model for predicting negative attitudes as a whole, and then to externally validate its performance on population and individual levels.

**Methods:**

938,866 Weibo posts with relevant keywords were downloaded, including 737,849 posts updated between 2009 and 2014 (*2009–2014 dataset*), and 201,017 posts updated between 2015 and 2020 (*2015–2020 dataset*). (1) For model development, based on 10,000 randomly selected posts from *2009 to 2014 dataset*, a human-based content analysis was performed to manually determine labels of each post (non-negative or negative attitudes). Then, a computer-based content analysis was conducted to automatically extract psycholinguistic features from each of the same 10,000 posts. Finally, a classification model for predicting negative attitudes was developed on selected features. (2) For model validation, on the population level, the developed model was implemented on remaining 727,849 posts from *2009 to 2014 dataset*, and was externally validated by comparing proportions of negative attitudes between predicted and human-coded results. Besides, on the individual level, similar analyses were performed on 300 randomly selected posts from *2015 to 2020 dataset*, and the developed model was externally validated by comparing labels of each post between predicted and actual results.

**Results:**

For model development, the F1 and area under ROC curve (AUC) values reached 0.93 and 0.97. For model validation, on the population level, significant differences but very small effect sizes were observed for the whole sample (*χ*^2^_1_ = 32.35, *p* < 0.001; Cramer’s V = 0.007, *p* < 0.001), men (*χ*^2^_1_ = 9.48, *p* = 0.002; Cramer’s V = 0.005, *p* = 0.002), and women (*χ*^2^_1_ = 25.34, *p* < 0.001; Cramer’s V = 0.009, *p* < 0.001). Besides, on the individual level, the F1 and AUC values reached 0.76 and 0.74.

**Conclusion:**

This study demonstrates the efficiency and necessity of machine learning prediction of negative attitudes as a whole, and confirms that external validation is essential before implementing prediction models into practice.

## Background

Negative attitudes towards suicide have strong negative health effects on suicidal people, including reduced access to quality health care and poorer psychological well-being (1–3). The mass media contributes to the dissemination of misinformation about negative attitudes towards suicide (4–7). If the characteristics of such misinformation can be understood, it may be possible to design targeted interventions to counter its negative influence and promote public mental health awareness. Many studies employed human coders to manually analyze suicide-related misinformation in traditional mass media (e.g., newspapers). For example, Flynn and colleagues investigated the portrayal of 60 cases of homicide-suicide in newspapers from England and Wales, and found homicide-suicide was commonly described with negative stereotypes (8). Creed and colleagues collected and coded articles about Robin William’s suicide from Canadian newspapers, and found a lack of necessary information that could help prevent suicide in 73% of articles (9). Sorensen and colleagues performed a thematic analysis of 78 suicide-related articles in Sri Lankan newspapers, and found the prevalence of biased news reporting (10). However, because of the sheer volume of mass media messages, it is difficult to efficiently screen for misinformation by human coders. Therefore, there is a dire need for automatic prediction of negative attitudes towards suicide, which may give us an efficient way to target misinformation among massive information or at least reduce the searching scope quickly for human coders.

As a representative of the new media, social media has introduced a new way to predict public attitudes towards mental health problems. Social media enables its millions of users to share their thoughts, feelings, and opinions with the public, and allows for viewing and downloading digital recordings of their publicly available online conversations. Therefore, by analyzing language use patterns of social media posts, many previous studies attempted to develop machine learning models for automatic prediction of misinformation regarding negative attitudes towards mental health problems, including depression, anxiety, and suicide (11–13). However, previous studies mainly focused on stigmatizing attitudes (negative stereotyping of suicidal people) and did not fully cover other forms of negative attitudes (without negative stereotyping of suicidal people), like dismissive, encouraging, cynical/indifferent, and disgusted attitudes associated with no stigmatizing expressions (6, 7, 14–16). Therefore, further studies are needed to incorporate varied forms of negative attitudes together and predict them as a whole.

In addition, before implementation into practice, external validation is needed to ensure that a prediction model is generalizable to new input data. External validation can be done by testing the model’s output in data that is not the same as the data used to create the model. However, in many previous studies, only internal validation was performed by using cross-validation techniques (11, 17, 18). Therefore, further studies are also needed to carry out additional external validation after the development of a prediction model.

To address these concerns, by analyzing a large-scale social media dataset (Sina Weibo, a Chinese social media site that is similar to Twitter), this paper aims to consider varied forms of negative attitudes together and develop a classification model for predicting negative attitudes as a whole, and then to externally validate the developed model on two different levels of granularity (population and individual levels). In this paper, negative attitudes refers to a set of emotions, beliefs, and behavioral intentions that may create negative outcomes for suicidal people.

## Methods

This study was approved by the Institutional Review Board at the Institute of Psychology, Chinese Academy of Sciences (protocol number: H15009). Informed consent was not obtained as this study solely focused on publicly available data and involved no personally identifiable data collection or analysis.

**Figure 1:**
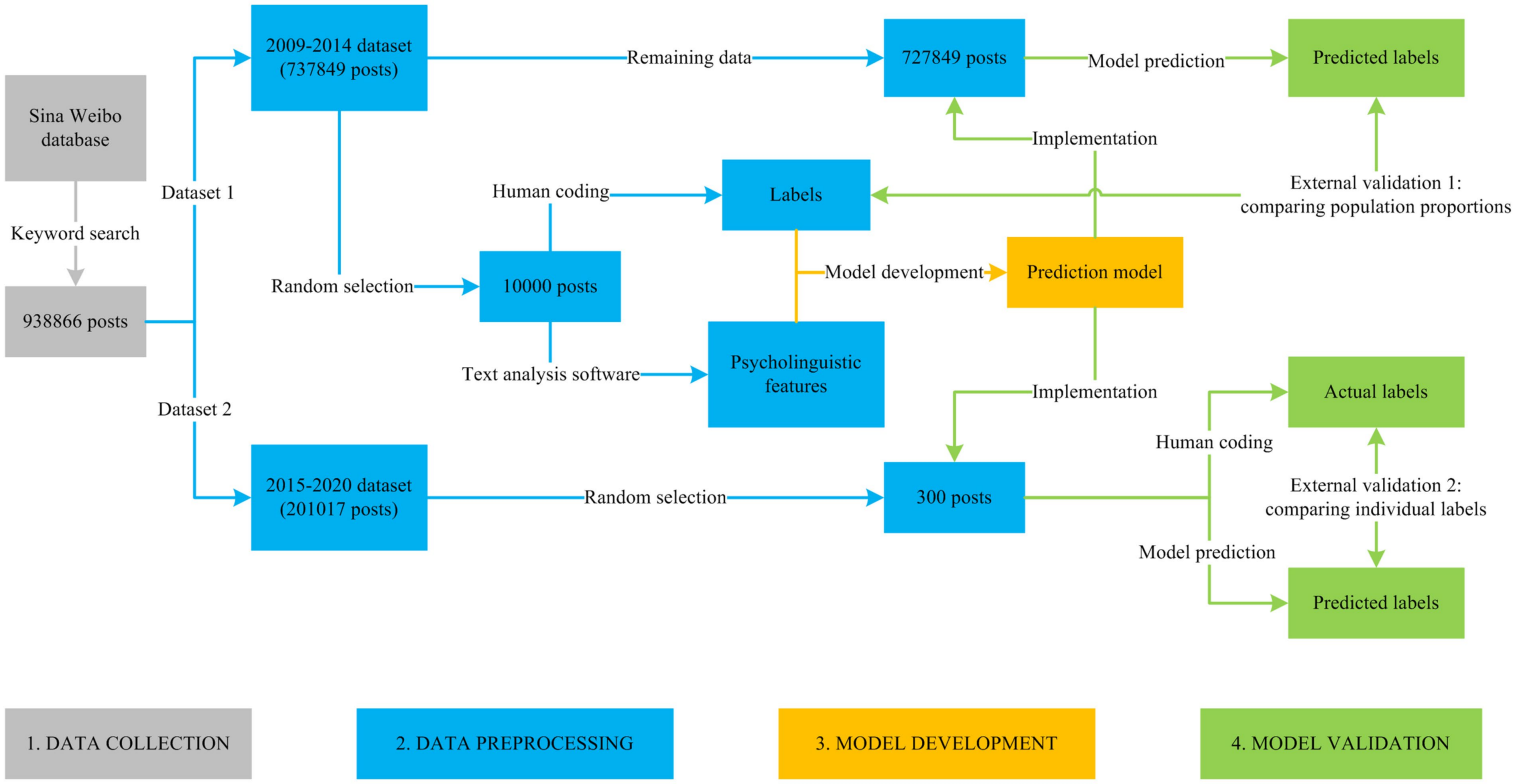
Research process.

This study consisted of four steps: (1) data collection, (2) data preprocessing, (3) model development, and (4) model validation (Figure 1).

### Data collection

Suicide-related Sina Weibo posts were identified and downloaded. In a previous paper, a Sina Weibo database consisting of 1,953,485 active users and theirs Weibo posts since registration was created (19). In specific, an initial user (seed user) was randomly selected, and a breadth-first search algorithm was utilized to search for all other users incorporated in the seed user’s social network. Then, the active users among them were identified based on their total number of posts and average number of posts per day, and all theirs Weibo posts since registration were downloaded via Application Programming Interface (API) to form the Sina Weibo database. After the creation of the database, according to a previous study (13), in order to identify as many suicide-related posts as possible, this study chose “suicide” (自杀) as the keyword to search the database and collected data in May 2020. Finally, a total of 938,866 posts with keywords were downloaded (men: 489138 posts; women: 449728 posts).

### Data preprocessing

After data collection, a data preprocessing was performed on raw data to prepare it for further analysis.

First, the downloaded data was divided into two subsets: (1) *2009–2014 dataset* (737,849 posts updated between 29 August 2009 and 31 December 2014; men: 390756 posts; women: 347093 posts), and (2) *2015–2020 dataset* (201,017 posts updated between 1 January 2015 and 28 May 2020; men: 98382 posts; women: 102635 posts).

Second, in *2009–2014 dataset*, a total of 10,000 posts were randomly selected from 737,849 posts (men: 5341 posts; women: 4659 posts). In order to develop a machine learning model for predicting negative attitudes as a whole, content analyses were performed on selected 10,000 posts to obtain values of predictors and predicted outcomes, respectively. Specifically, for one thing, to obtain values of predictors, a computer-based content analysis was performed by a text analysis software (Simplified Chinese version of Linguistic Inquiry and Word Count, SCLIWC) to extract psycholinguistic features from each post. SCLIWC is a reliable and valid text analysis tool for automatic estimation of word frequency in multiple psychologically meaningful categories (20). For another thing, to obtain values of predicted outcomes, a human-based content analysis was performed by two independent human coders to determine the label of each post (i.e., non-negative or negative attitudes).

### Model development

After data preprocessing, by using 10,000 posts randomly selected from *2009 to 2014 dataset*, a machine learning model was developed on selected psycholinguistic features to automatically predict the label of a given input data.

First, to improve prediction performance of the model, a subset of psycholinguistic features that were valid for differentiating between non-negative and negative attitudes were selected as key features for use in model construction.

In this paper, to avoid over-fitting of the model, the data used for feature selection remained independent of the data used for model training. Therefore, among 10,000 selected posts, a total of 2000 posts were randomly selected for feature selection and the remaining 8,000 posts were used for model training.

The process of feature selection was evaluated by 10-fold cross-validation, and key features were identified by three different feature selection methods. By using each feature selection method, all extracted psycholinguistic features were ranked by their importance for classification. Those ranked among the top-20 features by all three methods were considered as key features. In this paper, by using the Waikato Environment for Knowledge Analysis software (WEKA, version 3.9.6), three different feature selection methods were implemented to rank psycholinguistic features by their individual evaluations, including CorrelationAttributeEval (CAE), InfoGainAttributeEval (IGAE), and SVMAttributeEval (SVMAE). Specifically, methods of CAE, IGAE, and SVMAE evaluate the worth of each feature by measuring the correlation between it and class, measuring the information gain with respect to the class, and using an support vector machine classifier, respectively.

Second, by using the automated machine learning package for WEKA (Auto-WEKA, https://www.cs.ubc.ca/labs/algorithms/Projects/autoweka), a classification model was developed based on selected key features. The Auto-WEKA is a tool for automating algorithm selection (including 30 machine learning algorithms) and hypermeter optimization. After importing a dataset into WEKA, the Auto-WEKA can be run on it to automatically determine the best model and its parameters, and performs internal 10-fold cross-validation.

### Model validation

After model development, predictive performance of the classification model was externally validated on a large-scale dataset, which was independent from the dataset used to create the model. In this paper, the external validation was carried out on two different levels of granularity (population and individual levels).

First, on the population level, in *2009–2014 dataset*, the remaining 727,849 posts (737849–10,000 = 727,849) were used for external validation 1 (men: 385415 posts; women: 342434 posts). Specifically, the developed classification model was implemented on the remaining 727,849 posts to automatically predict the label of each post. Because the 10,000 human-coded posts used for creating the model were randomly selected from the *2009–2014 dataset*, it was assumed that there should be no significant differences in proportions of negative attitudes between predicted results (727,849 posts) and human-coded results (10,000 posts).

Second, on the individual level, in *2015–2020 dataset*, a total of 300 posts were randomly selected and analyzed by two independent human coders to determine the label of each post. Then, the developed classification model was implemented on the selected 300 posts to automatically predict the label of each post. The differences in labels of each post between predicted and actual results were examined for external validation 2.

## Results

### Human coding

The Cohen’s *k* coefficients for attitudes reached the level of near perfect agreement (10,000 posts from *2009 to 2014 dataset*: 0.92; 300 posts from *2015 to 2020 dataset*: 0.95) (21). Results of human coding were shown in Table 1.

**Table 1:** Results of human coding.

	2009–2014 dataset	%	2015–2020 dataset	%
All	10,000		300	
Non-negative attitudes	5,966	59.66%	176	58.67%
Negative attitudes	4,034	40.34%	124	41.33%
Men	5,341		143	
Non-negative attitudes	3,216	60.21%	93	65.03%
Negative attitudes	2,125	39.79%	50	34.97%
Women	4,659		157	
Non-negative attitudes	2,750	59.03%	83	52.87%
Negative attitudes	1909	40.97%	74	47.13%

### Feature selection

The top-20 ranked psycholinguistic features by three different feature selection methods were shown in Table 2, respectively. As a result, a total of six psycholinguistic features were selected as key features, including *adverbs*, *positive emotion*, *assent*, *health*, *swear*, and *2nd pers singular*.

**Table 2:** The top 20 ranked psycholinguistic features.

	CorrelationAttributeEval	InfoGainAttributeEval	SVMAttributeEval
1	***Adverbs***	***Adverbs***	***Swear***
2	***Positive emotion***	Total function words	***Positive emotion***
3	Total pronouns	Personal pronouns	***Health***
4	Total function words	***Health***	Signal words for past tense
5	Personal pronouns	Total pronouns	***Adverbs***
6	***Assent***	Word count	Motion
7	***Health***	***Assent***	Sad
8	Semicolons	***Positive emotion***	***2nd pers singular***
9	1st pers. singular	1st pers. singular	Sexual
10	Social processes	Semicolons	Common verbs
11	***Swear***	Signal words for continuous tense	Anger
12	Word count	Exclamation marks	Time
13	Sad	Social processes	Dashes
14	Signal words for tenses	***Swear***	Apostrophes
15	Signal words for continuous tense	Signal words for tenses	***Assent***
16	Motion	***2nd pers singular***	3rd pers. plural
17	***2nd pers singular***	Biological processes	Negations
18	Affective processes	3rd pers. singular	Relativity
19	Biological processes	Affective processes	Question marks
20	Exclusive	Humans	Religion

Bold and italics indicate key features.

### Model development

A classification model using the C4.5 algorithm was developed on six key features. The classification performance of the C4.5 model was evaluated using precision, recall, F1, and area under ROC curve (AUC). The weighted-average F1 and AUC values reached 0.93 and 0.97, respectively (Table 3). Since model training outcomes did not indicate relationships between psycholinguistic features and predicted labels, a logistic regression analysis was performed to explore such relationships (Table 4).

**Table 3:** Performance of the developed C4.5 model.

	Precision	Recall	F1	Area under ROC curve
Class 1: non-negative attitudes	0.92	0.96	0.94	0.97
Class 2: negative attitudes	0.94	0.88	0.91	0.97
Weighted-average	0.93	0.93	0.93	0.97

**Table 4:** Relationships between psycholinguistic features and predicted labels.

Predicted labels	Psycholinguistic features	β
Non-negative attitudes (1) vs. Negative attitudes (2)	Adverbs	5.75
	Positive emotion	12.48
	Assent	2.98
	Health	−12.56
	Swear	21.07
	2nd pers. singular	12.27

### Model validation

First, on the population level, significant differences but very small effect sizes were observed between predicted results (727,849 posts) and human-coded results (10,000 posts) for the whole sample (*χ*^2^_1_ = 32.35, *p* < 0.001; Cramer’s V = 0.007, *p* < 0.001), men (*χ*^2^_1_ = 9.48, *p* = 0.002; Cramer’s V = 0.005, *p* = 0.002) and women (*χ*^2^_1_ = 25.34, *p* < 0.001; Cramer’s V = 0.009, *p* < 0.001) (Table 5).

**Table 5:** Comparison of predicted and human-coded results (2009–2014 dataset).

	Predicted	%	Human-coded	%
All	727,849		10,000	
Non-negative attitudes	454,424	62.43%	5,966	59.66%
Negative attitudes	273,425	37.57%	4,034	40.34%
Men	385,415		5,341	
Non-negative attitudes	239,996	62.27%	3,216	60.21%
Negative attitudes	145,419	37.73%	2,125	39.79%
Women	342,434		4,659	
Non-negative attitudes	214,428	62.62%	2,750	59.03%
Negative attitudes	128,006	37.38%	1909	40.97%

Second, on the individual level, between predicted and actual results (300 posts), the weighted-average F1 and AUC values reached 0.76 and 0.74, respectively (Table 6).

**Table 6:** Comparison of predicted and actual results (2015–2020 dataset).

	Precision	Recall	F1	Area under ROC curve
Class 1: non-negative attitudes	0.79	0.80	0.80	0.74
Class 2: negative attitudes	0.71	0.70	0.71	0.74
Weighted-average	0.76	0.76	0.76	0.74

## Discussion

### Principal results

This paper incorporated varied forms of negative attitudes together and developed a classification model for predicting negative attitudes in social media texts, and then externally validated its predictive performance on two different levels of granularity (population and individual levels), using a large-scale dataset. Results of this paper demonstrate the efficiency and necessity of machine learning prediction of negative attitudes as a whole, and confirm that external validation is essential before implementing prediction models into practice.

First, it is necessary to consider varied forms of negative attitudes together to better understand the nature of public reactions to suicide. In this paper, human-coded results showed that, among suicide-related posts, the prevalence of negative attitudes towards suicide was approximately around 40%, which is obviously higher than the findings of previous studies that focused on limited forms of negative attitudes (23% ~ 35%) (15, 16). It means that, on Chinese social media, negative attitudes towards suicide may be actually much more prevalent than previously estimated. According to the Healthy China Action Plan (2019–2030), by 2030, health literacy of the entire Chinese population is expected to be greatly improved (22). Therefore, to promote public mental health awareness as planned, mental health professionals may be under greater pressure than ever before, and there is a dire need for innovative approaches to efficiently promote public reactions to suicide.

Second, the method of psycholinguistic analysis provides insights into the language use patterns of negative expressions towards suicide, and facilitates automatic prediction of negative attitudes in social media texts. Results of this paper showed that expressions of negative attitudes towards suicide were associated with increased use of words related to adverbs (e.g., very), positive emotion (e.g., nice), assent (e.g., ok), swear (e.g., fuck), and 2nd pers. singular (e.g., you); and decreased use of words related to health (e.g., clinic). According to previous studies, increased use of swear-related words reflects enhanced emotional-related impulsivity and frustration, and therefore are commonly considered as an indicator of negative affect (23–25). Besides, more frequent use of health-related words indicates people are more concerned about health problems (26). Therefore, in this paper, decreased use of health-related words may imply a view that suicide is not worth paying attention to, and was related to negative attitudes. Moreover, it is worthy to note here that increased use of words related to positive emotion and assent are positive predictors for emotional support in previous studies (27, 28). However, in this paper, increased use of words indicating positive emotion and assent were related to negative attitudes towards suicide, which may be due to the prevalence of normalization or glorification of suicide (29, 30). With the help of psycholinguistic analysis techniques, compared with results of other similar studies (F1 values: 0.62 ~ 0.86) (11, 13, 17), the developed machine learning model performed well in classifying non-negative and negative expressions (F1 value: 0.76 ~ 0.93). It means that understanding the differences in language use patterns between non-negative and negative expressions would make the features used in machine-learning models more understandable for end user and make machine learning prediction more precise. Furthermore, the use of machine learning approaches for automatic prediction of negative attitudes provide us an efficient way to reduce the workload of human coders for searching misinformation. For example, in this paper, the developed classification model was implemented on 727,849 unlabeled posts, and then automatically labeled 273,425 of them as negative attitudes. It means that 62.43% of the workload of human coders can be reduced quickly (62.43% = (727849−273,425)/727,849).

Third, external validation of machine learning prediction models is crucial before clinical application. Without the process of external validity, it is unclear about the extent to which the prediction performance of the developed model can be generalized to other situations. In this paper, by using an internal cross-validation technique, a machine learning model was developed, and the weighted-average F1 and AUC values reached a nearly perfect level of prediction performance (internal validity: 0.93 and 0.97). However, when the developed model was implemented to the new data that is not the same as the data used to develop the model, the weighted-average F1 and AUC values dropped to a satisfactory level of prediction performance (external validity: 0.76 and 0.74). It means that, although similar studies have already performed an internal validity of developed mental health machine learning models and have achieved good predictive performance, additional external validity tests are still needed to ensure that a prediction model is generalizable to new input data other than those in the derivative cohort. Results of the external validity may show us the actual predictive performance of developed models under realistic conditions, and provide implications for making large-scale automatic and non-intrusive forecasting of public attitudes towards suicide. Findings suggest that, under realistic conditions, population-based prediction (predicting proportions of negative attitudes) may be considerably more effective than prediction that targets individuals (predicting labels of each post).

### Limitations

Limitations existed in this study. First, because the number of posts is extremely unbalanced and limited among varied forms of negative attitudes, machine learning models for predicting specific forms of negative attitudes cannot be developed in this paper. Second, this paper mainly focused on Sina Weibo users, who are not representative of Chinese populations or all Chinese social media users. Therefore, the findings may not be applicable to other non-users. Third, this paper focused on a single social media platform (Sina Weibo) and only analyzed Chinese social media posts, so the findings of this paper may have limited generalizability. Fourth, since the Auto-WEKA package can only perform automatic algorithm selection on 30 classical machine learning algorithms, it is not known if using the latest machine learning algorithms (e.g., deep learning models and large language models) will improve the predictive performance of the classification model.

## Conclusion

This paper demonstrates the efficiency and necessity of machine learning prediction of negative attitudes as a whole, and confirms that external validation is essential before implementing prediction models into practice. Findings suggest that, under realistic conditions, population-based prediction may be considerably more effective than prediction that targets individuals.

## Data availability statement

The datasets presented in this article are not readily available because the raw data cannot be made public (if necessary, feature data can be provided). Requests to access the datasets should be directed to AL, angli@bjfu.edu.cn.

## Ethics statement

The studies involving humans were approved by Institutional Review Board at the Institute of Psychology, Chinese Academy of Sciences. The studies were conducted in accordance with the local legislation and institutional requirements. Written informed consent for participation was not required from the participants or the participants’ legal guardians/next of kin because this study solely focused on publicly available data and involved no personally identifiable data collection or analysis.

## Author contributions

AL: Conceptualization, Investigation, Formal analysis, Methodology, Writing – original draft.
